# Swiss residents' speciality choices – impact of gender, personality traits, career motivation and life goals

**DOI:** 10.1186/1472-6963-6-137

**Published:** 2006-10-23

**Authors:** Barbara Buddeberg-Fischer, Richard Klaghofer, Thomas Abel, Claus Buddeberg

**Affiliations:** 1 Department of Psychosocial Medicine, Zurich University Hospital, Haldenbachstrasse 18, CH-8091 Zurich, Switzerland; 2 Department of Health Research, University of Bern, Niesenweg 6, CH-8013 Bern, Switzerland

## Abstract

**Background:**

The medical specialities chosen by doctors for their careers play an important part in the development of health-care services. This study aimed to investigate the influence of gender, personality traits, career motivation and life goal aspirations on the choice of medical speciality.

**Methods:**

As part of a prospective cohort study of Swiss medical school graduates on career development, 522 fourth-year residents were asked in what speciality they wanted to qualify. They also assessed their career motivation and life goal aspirations. Data concerning personality traits such as sense of coherence, self-esteem, and gender role orientation were collected at the first assessment, four years earlier, in their final year of medical school. Data analyses were conducted by univariate and multivariate analyses of variance and covariance.

**Results:**

In their fourth year of residency 439 (84.1%) participants had made their speciality choice. Of these, 45 (8.6%) subjects aspired to primary care, 126 (24.1%) to internal medicine, 68 (13.0%) to surgical specialities, 31 (5.9%) to gynaecology & obstetrics (G&O), 40 (7.7%) to anaesthesiology/intensive care, 44 (8.4%) to paediatrics, 25 (4.8%) to psychiatry and 60 (11.5%) to other specialities. Female residents tended to choose G&O, paediatrics, and anaesthesiology, males more often surgical specialities; the other specialities did not show gender-relevant differences of frequency distribution. Gender had the strongest significant influence on speciality choice, followed by career motivation, personality traits, and life goals. Multivariate analyses of covariance indicated that career motivation and life goals mediated the influence of personality on career choice. Personality traits were no longer significant after controlling for career motivation and life goals as covariates. The effect of gender remained significant after controlling for personality traits, career motivation and life goals.

**Conclusion:**

Gender had the greatest impact on speciality and career choice, but there were also two other relevant influencing factors, namely career motivation and life goals. Senior physicians mentoring junior physicians should pay special attention to these aspects. Motivational guidance throughout medical training should not only focus on the professional career but also consider the personal life goals of those being mentored.

## Background

The medical specialties chosen by doctors for their careers play an important role in the development of health-care services. There are some recent studies of how different medical specialities are perceived or how choices are made [1-3]. Several determinants have been identified: the feminisation of medicine, lifestyle, specialist status and the prospect of future income, as well as the structural conditions of the various residency programmes. Most studies addressed medical students, not residents. As far as we know there appear to be only a few studies on personality traits, career motivation and personal life goals as influencing factors on speciality choice [4].

### Feminisation

Since the 1990s, more than half of the medical school graduates in Western countries have been women [5-8]. Although female physicians tend to specialise almost to the same degree, they enter other specialties than their male colleagues [9-12]. Especially in surgical specialities, female physicians are under-represented [13]. Gender differences in speciality choice can partly be explained as a function of socialisation [14-16], but also by structural operating barriers or closure mechanisms within specific fields [17-19].

### Lifestyle

Several studies have reported that a so-called controllable lifestyle has become a determinant in physicians' speciality selection criteria [3,20-22]. The following characteristics of a controllable lifestyle have been defined: personal time free of practice requirements for leisure, family, and non-vocational pursuits and control of total weekly hours spent on professional responsibilities. For female physicians the prospect of combining their professional career with family responsibilities is a key issue in the process of speciality choice or changing the speciality to which they initially aspired to [16].

### Status and income

Prestige within the medical profession, social status and income also play their role in the decision in favour of a medical speciality [23,24]. In some studies, students reported their student debt as one of the factors influencing their career choices [25,26]. Students with large debts tended to choose surgical specialities more often and were less likely to choose primary care.

### Residency programmes

The application and selection procedures for residency programmes, the length, quality and structure of the programme, work schedules, mentorship, annual vacations are also factors which are considered when choosing a speciality [18,19,24,27,28].

Most studies investigated only one or two of the factors identified as influencing speciality choice. One has to consider, however, that Swiss studies addressing issues of speciality choice are lacking to date.

**Figure 1:**
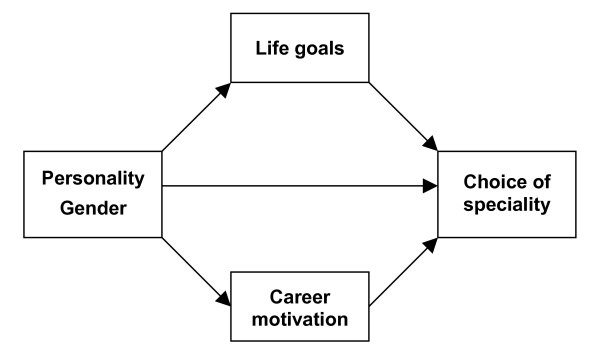
Conceptual framework of the study.

The aims of this study were to investigate (1) the development of the residents' speciality choices since graduating from medical school, and the differences compared to the speciality distribution of working doctors, and (2) what factors influence the young doctors' speciality choices. As shown in Figure 1, we hypothesised that gender and personality traits have an impact on speciality choice, and that career motivation as well as life goals have an influence, too. The present paper aimed to examine these hypotheses.

## Methods

### Study design, sample development, and study sample

The present study is part of an ongoing prospective survey of a cohort of graduates of the three medical schools in German speaking Switzerland, beginning in 2001 (T_1_). Of the 1004 registered final-year students, 715 (71%) participated in the first assessment (T_1_, in 2001) [15]. Subjects were re-evaluated after two years in 2003 (T_2_) [18,19]. The present paper refers to results of the third assessment (T_3_), conducted in the fourth year of residency (in 2005). Table 1 shows the *sample development *from T_0 _(questionnaires sent to all registered graduates at the medical schools of Basel, Bern, and Zurich) to T_1_, T_2_, and T_3 _for participants, non-participants and dropouts. There are no significant differences between the 193 dropouts (T_1 _– T_3_) and the 522 subjects participating at the third measurement with regard to socio-demographic data, personality traits, and career-related variables at T_1_.

Of these 522 residents there were 281 females (53.8%) and 241 males (46.2%). The mean age was 31.3 years (SD 2.4 y, range 27 – 46 y). Of the residents 428 (82.0%) had a stable partnership, of whom 125 were married (65 females and 60 males). Only 26 (9.3%) of the females, but 36 (14.9%) of the males had own children (p = 0.03). 94.7% worked full-time, 5.3% part-time. The mean working hours per week were 55.4 hrs (SD 8.2 hrs, range 30 – 80 hrs).

### Speciality training and residencies in Switzerland

There are 43 registered speciality qualifications in Switzerland. Most of the specialities require at least a six-year residency, and a final speciality qualification exam. One major problem with most of the speciality training courses is the lack of structured and time-limited residency programmes. Young doctors usually only get contracts for a year, i.e. they have to arrange and organize their residency posts every one to two years.

### Instruments

The main characteristics of the applied instruments are given in Table 2. All instruments are self-assessment scales. In the following, it is described what constructs are measured by the instruments:

• *Questions concerning socio-demographic data and choice of medical speciality*

• *Sense of Coherence Scale, SOC-13 *[29], is a measure of a person's resistance to stress and his/her ability to manage stress (measure of traits, stability in this study T_1 _– T_3_: 0.56).

• *Rosenberg-Self-Esteem-Scale, RSE *[30], assesses general self-esteem and includes items that express a general favourable or unfavourable attitude towards oneself (measure of traits).

• *Personal Attributes Questionnaire*, *GE-PAQ*, German Extended Personal Attributes Questionnaire [31], is a self-rating instrument for the assessment of gender-role orientation (measure of traits). The *Instrumentality (PAQ-I) *scale contains instrumental traits (e.g. 'independent', 'decisive') that are considered to be socially desirable to some degree in both sexes but stereotypically more characteristic of males. The *Expressiveness (PAQ-E) *scale contains so-called 'feminine' items that describe socially desirable expressive, communal traits (e.g. 'helpful') that are stereotypically more characteristic of females.

• *Career Motivation Questionnaire, CMQ *[32], consists of 3 scales (measure of traits): *Intrinsic Career Motivation CMQ-I *(i.e. enjoyment of and interest in professional activities) (stability in this study T_1 _– T_3_: 0.57), *Extrinsic Career Motivation CMQ-E *(i.e. striving for promotion, income, prestige) (stability 0.58) and *Extraprofessional Concerns CMQ-EC *(i.e. prioritising family, convenient working hours, job security) (stability 0.60).

• *Life Goals Questionnaire, GOALS *[33], assesses 24 general, long-term life goals pertaining to six major life domains (measure of traits): *intimacy *(close relationships based on mutual trust and affection), *affiliation *(spending time with other people, common activities), *altruism *(acting for the welfare of others), *power *(asserting oneself, seeking social status), *achievement *(improving on oneself, meeting standards), and *variation *(seeking new experiences and excitement). Each goal is rated in regard to the *importance *(How important is it for you to reach this goal in your lifetime?). Importance ratings indicate which goals are desirable and valuable for the person and indicate the strength of his/her commitment to a goal.

### Statistical analyses

All analyses were carried out with SPSS for windows, release 12.0. Descriptive statistics were given in terms of counts and percentages, means and standard deviations respectively. Gender-different speciality choice was tested by Chi^2^-test. Study hypotheses were tested by univariate analyses of variance, followed by Scheffé-tests, and multivariate analyses of variance and covariance.

## Results

*Residents' speciality choices at T*_3_: Of the 522 physicians participating at the third assessment, 83 (15.9%) had not yet decided in which speciality they wanted to qualify. The remaining 439 residents had decided to qualify in the following specialities: 45 (8.6%) in primary care, 126 (24.1%) in internal medicine (including all sub-specialities of internal medicine), 68 (13.0%) in surgical disciplines, 31 (5.9%) in gynaecology & obstetrics, 40 (7.7%) in anaesthesiology and intensive care, 44 (8.4%) in paediatrics, 25 (4.8%) in psychiatry, and 60 (11.5%) in other specialities (such as dermatology, ENT, neurology, ophthalmology, radiology). The participants had a list of all 43 officially acknowledged medical specialities in Switzerland, marked with a code number, which they could fill in answering the question concerning the speciality choice.

**Figure 2:**
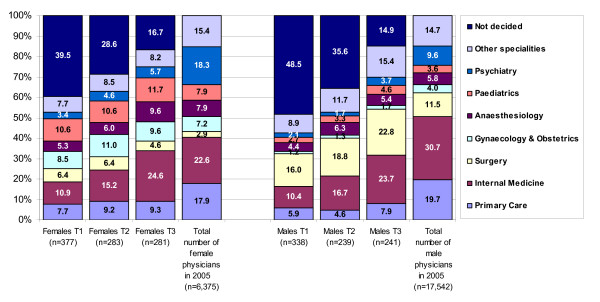
Study residents' speciality choices over time (T_1 _– T_3_) and speciality distribution of working doctors (2005).

*The development of residents' speciality choices *is shown in Figure 2. At the end of medical school 60% of the female and 51% of the male graduates had made their speciality choice. In their second year of residency, 71% of the female and 64% of the male residents had decided, and in the fourth year of residency, 83% of the female and 84% of the male participants named their speciality aspired to. The distribution of the different specialities within the gender group did not change much between the three measurements. However, the distribution of the specialities aspired to by the study participants is different from the *speciality distribution represented by the working doctors *who hold a speciality qualification. Compared to all working female specialists, there are significantly fewer female residents aspiring to become primary care physicians and psychiatrists; also the group of other specialities is smaller. It might be that some of the still undecided residents will choose one of those specialities later on. Looking at the male participants, fewer residents want to become primary care physicians, internists or psychiatrists but significantly more want to go into surgical disciplines compared to working male doctors.

The *speciality choice depending on gender *in the study sample at T_3 _is listed in Table 3. Male residents more often chose surgical specialities, whereas females decided on paediatrics, gynaecology & obstetrics (G&O), and anaesthesiology. In the group of other specialities there was no relevant gender-different distribution.

*Residents' characteristics depending on speciality aspired to *are shown in Table 4. Participants choosing surgical specialities or anaesthesiology comparably show the highest scores for sense of coherence, self-esteem and instrumentality; psychiatry residents give low scores on these three personality scales. With regard to the scores for expressiveness there are no significant differences between the groups. Physicians aspiring to surgical specialities have comparatively high values for intrinsic and extrinsic career motivation, but low values for extraprofessional concerns. Primary care physicians and psychiatrists rate extraprofessional concerns comparatively high. The life goal 'intimacy' is especially important for G&Os. Physicians in the surgical specialities as well as in G&O attach particular importance to 'power'. Future primary care physicians assess the life goal 'achievement' lowest. Subjects pursuing internal medicine show medium level values on all scales.

The multivariate analyses of the influence of gender, personality traits, career motivation and importance of life goals is shown in Table 5. The effect of gender remained significant after controlling for personality traits, career motivation and life goals as covariates. The same does not apply to the influence of personality traits on the speciality choice after controlling for career motivation and life goals. In other words, there are no direct significant influences of personality traits on the speciality choice. This means that career motivation and life goals can be considered as mediator variables.

## Discussion

The present study is part of an ongoing prospective survey of a cohort of Swiss medical school graduates. Subjects included in the study are fourth-year residents in different medical speciality fields. The aims of the study are to examine (1) *the development of the residents' speciality choices *since graduating from medical school and (2) *what factors influenced their choices*.

*Development of the residents' speciality choices: *at the end of medical school a considerable number of students have not yet developed precise ideas as to which speciality they want to work in. The main reason is lack of clinical experience. During the first and second years of post-graduate training they gain insight into various specialities, which makes it easier for them to make their decision. Some residents change the speciality they primarily aspired to, but not so many do. Nor is a marked shift away from one speciality towards another evident. Compared to the working doctors, significantly fewer young doctors aspire to become primary care physicians (PCPs) or psychiatrists. In the competition-based health care systems of Switzerland and the other German-speaking countries the professional prestige, social status, and income of other specialists is much higher than those of PCPs and psychiatrists. As long as the current health policy does not create better professional conditions, the shift away from PC and psychiatry will continue. If this trend prevails for some time, there will be a shortage of doctors providing basic somatic and mental health care, especially in rural areas.

*Factors influencing speciality choice: *As hypothesized, we found *gender different speciality choices*, female doctors being over-represented in specialities like gynaecology & obstetrics (G&O), paediatrics, and anaesthesiology and male doctors in surgical specialities. Similar results are reported in other studies [11-13,17,34]. Although G&O entails long hours and a heavy surgical workload, a growing number of women choose this speciality. Women are interested in surgical specialities, but often experience gender-relevant exclusion mechanisms in other surgical fields [17,35]. The marked gender shift in G&O is due to the growing attitude, starting in the late 1980s, that women should be treated by female physicians [36]. Paediatrics, the other speciality mainly chosen by women, is also a speciality in which gender schemas play a certain role [14]. This gender-distinct speciality choice was already found when the participants were in the last year of medical school [15]. The reasons for an increasing number of female doctors choosing anaesthesiology might be manifold: anaesthesiology is a professionally prestigious speciality like surgery but not as competitive, it covers a broad medical spectrum and offers good options for part-time work and good promotion prospects, all factors appreciated by females. Whether the decisive role of gender is based on internalised gender roles or whether there are open or masked deterrents cannot be distinguished by this study.

*Personality, career motivation, life goals and speciality choice: *According to our assumption, we found that gender, personality, career motivation and life goals have an impact on speciality choice. Petrides and McManus [1] described a mapping of medical careers based on the typology found by Holland in careers in general. Holland's theory [37] suggests that careers can be organised into six broad types, which can be represented around a hexagon, known by the acronym RIASEC, standing for 'Realistic', 'Investigative', 'Artistic', 'Social', 'Enterprising' and 'Conventional'. They also referred to the 'Things↔People' and 'Ideas↔Data' dimensions proposed by Prediger [38] which can be underpinned to Holland's typology. Both models use the attribution of the medical specialities based on the characteristics of their professional activities. We suppose that residents choose a medical speciality in which they can conduct their professional activities corresponding to their special personality traits, career motivation, and life goals aspired to. The residents in *surgical specialities *in our study were characterised by high values for instrumentality, intrinsic and extrinsic career motivation, 'power' and 'achievement' as life goals. These attributes and attitudes are mainly 'Things/technique-oriented' according to Prediger and can be assigned to Holland's realistic career type. Participants choosing *anaesthesiology and intensive care *assessed themselves almost as highly instrumental as doctors in surgery. They can also be mapped to the realistic type. Contrary to the study by Petrides & McManus [1], the G&O residents in our sample revealed characteristics of 'People Orientation', matching the social type, a fact which might be influenced by the high number of females in this group. They stated high expressiveness and life goals aiming at satisfying social relationships. *Paediatricians *in our study, mainly females, showed similar characteristics as the G&Os. *Psychiatrists *differed from all the other specialities by rating the extraprofessional concerns highest, while expressiveness and altruism were not values very high. They could not easily be assigned to one of the RIASEC types. The same applies for *primary care physicians*. They showed characteristics of 'People Orientation' (high extraprofessional concerns and altruism), and rated in the medium range in most of the other aspects. The internists were allocated to the investigative type by Petrides & McManus [1], a type in which patient-relationships and diagnostic investigations play their part. This might also apply to the *internists *in our study; they described themselves as empathetic (adequate expressiveness), but also instrumentally-oriented. The RIASEC mapping of medical specialities did not consider career motivation and life goals but focused only on personality traits. Our results indicate that career motivation and life goals are even more important for the speciality choice than personality traits.

In summary, the results of our study indicate that gender plays a decisive role in speciality choice, while the influence of personality declines after controlling for career motivation and life goals. The feminisation of medicine and especially of some specialities can be expected to lead to fundamental changes in the medical system. One can assume that the style of leadership in hospitals will change: As long as men are department heads, a "command and control style of managing others" will predominate, while women tend towards "interactive leadership" [39]. Other aspects concern employment: more part-time jobs have to be provided for women doctors with family obligations. However, the feminisation also carries the risk of the danger of vertical and horizontal gender segregation [40]: female physicians often spend more time with patients while male doctors look at what is more advantageous for their career, such as laboratory work, developing research projects and writing papers. These differences in working attitudes result in gender-different career opportunities: males taking over leadership positions and females looking after their patients' needs.

## Conclusion

The speciality choice of the new generation of doctors is a matter of concern for the health care system in Switzerland. Ideally, as many doctors should practise in a speciality as are needed to cover the population's health care. As the statistics of the Swiss Medical Association and our study data show, a lack of primary care physicians as well as of psychiatrists will occur in the next decade. The question arises whether incentives, mainly financial, will encourage more young doctors to choose to become primary care physicians or psychiatrists, or whether access to the speciality qualification programmes and to the allocation of private practice licenses should be regulated by health policy.

There should be an acknowledgment of the distinctive features of female physicians' careers. Workplace conditions should allow women doctors, if they want, to combine pursuing a prestigious career in medicine with having a family. There is a need to promote and encourage their instrumental traits and extrinsic career motivation. Motivational guidance by senior physicians throughout medical training should not only focus on the professional career but should also consider the personal life goals of those being mentored.

## Competing interests

The author(s) declare that they have no competing interests.

## Authors' contributions

All authors designed the study. BBF was responsible for data acquisition, RK conducted the statistical analyses. BBF, RK, TA and CB contributed to the interpretation of data, BBF drafted the manuscript versions, RK, TA and CB revised the drafts critically for important intellectual content. All authors gave final approval to the version to be published.

## Pre-publication history

The pre-publication history for this paper can be accessed here:



## Figures and Tables

**Table 1:** Development of the study sample

	Sample addressed T_0_(2001) n (%)	Non-participants n (%)	Study sample **T_1_**(2001) n (%)	Drop-outs T_1 _– T_2 _ n (%)	Study sample **T_2_**(2003) n (%)	Drop-outs T_1 _– T_3 _ n (%)	Study sample **T_3_**(2005) n (%)
*Gender*							
female	487 (49)	108 (37)	379 (53)	96 (50)	283 (54)	98 (51)	281 (54)
male	517 (51)	181 (63)	336 (47)	97 (50)	239 (46)	95 (49)	241 (46)
*Age in years*							
mean			27.4	27.6	29.3	31.5	31.3
range			23 – 44	23 – 44	26 – 44	29 – 48	27 – 46

Total	**1004**	289	**715**	193	**522**	193	**522**

**Study sample**			**715** (100%) (71% of 1004)		**522 ** (73% of 715) (52% of 1004)		**522 ** (73% of 715) (52% of 1004)

**Table 2:** Characteristics of the applied instruments

**Dimensions** **and scales**	Number of items	Method of item scoring (Likert-scales)	Method of scale scoring	Reference value (mean) female	Reference value (mean) male	Reliability	Reliability in this study	Stability in this study T_1 _– T_3_
**Personality factors (T_1_)**								
Sense of coherence (SOC)	13	1 (low) – 7 (high)	Sum score divided by number of items	4.96	5.18	0.85	0.84	0.56
Rosenberg Self-esteem	10	1 (low) – 4 (high)	Sum score/number of items	2.23	2.31	0.88	0.85	
^1^PAQ Instrumentality	8	1 (low) – 6 (high)	Sum score/number of items	3.50	3.75	0.72	0.74	
^1^PAQ Expressiveness	8	1 (low) – 6 (high)	Sum score/number of items	4.63	4.38	0.75	0.74	
**Career-related factors (T_3_)**								
^2^CMQ Intrinsic career motivation	8	1 (low) – 7 (high)	Sum score/number of items	5.96		0.70	0.68	0.57
^2^CMQ Extrinsic career motivation	8	1 (low) – 7 (high)	Sum score/number of items	4.17		0.76	0.69	0.58
^2^CMQ Extra-professional concerns	8	1 (low) – 7 (high)	Sum score/number of items	4.30		0.72	0.74	0.60
**Importance of life goals (T_3_)**								
Intimacy	4	1 (low) – 5 (high)	Sum score/number of items	4.60		0.60	0.76	
Affiliation	4	1 (low) – 5 (high)	Sum score/number of items	3.47		0.82	0.85	
Altruism	4	1 (low) – 5 (high)	Sum score/number of items	3.55		0.76	0.82	
Power	4	1 (low) – 5 (high)	Sum score/number of items	2.73		0.85	0.83	
Achievement	4	1 (low) – 5 (high)	Sum score/number of items	4.00		0.68	0.75	
Variation	4	1 (low) – 5 (high)	Sum score/number of items	3.33		0.81	0.76	

^1 ^PAQ Personal Attributes Questionnaire

^2 ^CMQ Career Motivation Questionnaire

**Table 3:** Frequency distribution of speciality aspired to shown by gender at T_3 _(n = 522)

**Speciality**	**Females (n = 281)** **n (%)** **adjusted standardised residual**	**Males (n = 241)** **n (%)** **adjusted standardised residual**
Primary Care (n = 45)	26 (9.3) 0.6	19 (7.9) -0.6
Internal medicine (n = 126)	69 (24.6) 0.2	57 (23.7) -0.2
Surgical specialities (n = 68)	13 (4.6) -6.2	55 (22.8) 6.2
Gynaecology & Obstetrics (n = 31)	27 (9.6) 3.8	4 (1.7) -3.8
Anaesthesiology (n = 40)	27 (9.6) 1.8	13 (5.4) -1.8
Paediatrics (n = 44)	33 (6.3) 2.9	11 (4.6) -2.9
Psychiatry (n = 25)	16 (5.7) 1.0	9 (3.7) -1.0
Other specialities (n = 60)	23 (8.2) -2.6	37 (15.4) 2.6
Not decided (n = 83)	47 (16.7) 0.6	36 (14.9) 0.6

Chi-square = 65.14, df = 8, p < 0.001

**Table 4:** Means and standard deviations of personality traits, career motivation and importance of life goals according to speciality fields (n = 522)

**Scale**	**PC ** n = 45 Mean (SD)	**IM** n = 126 Mean (SD)	**SG** n = 68 Mean (SD)	**GO** n = 31 Mean (SD)	**AN** n = 40 Mean (SD)	**PAED** n = 44 Mean (SD)	**PSY** n = 25 Mean (SD)	**OS** n = 60 Mean (SD)	**ND** n = 83 Mean (SD)	**p**	**Scheffé-test**
**Personality traits**											
SOC Sense of Coherence	5.03 (0.73)	4.99 (0.84)	5.35 (0.82)	5.03 (0.80)	5.36 (0.77)	4.91 (0.93)	4.85 (1.03)	5.21 (0.83)	4.95 (0.77)	0.011	PSY, PAED, ND < SG, AN
RSE Rosenberg-Self-Esteem	2.42 (0.38)	2.27 (0.48)	2.48 (0.42)	2.30 (0.37)	2.50 (0.55)	2.31 (0.40)	2.27 (0.62)	2.44 (0.39)	2.33 (0.51)	0.037	IM, PSY < SG, AN
PAQ^1 ^Instrumentality	4.03 (0.65)	4.11 (0.63)	4.63 (0.55)	3.98 (0.73)	4.42 (0.69)	4.14 (0.67)	3.89 (0.73)	4.34 (0.59)	4.00 (0.75)	<0.001	PSY, GO, PC, ND < OS < AN < SG IM, PAED < SG, AN
PAQ^1 ^Expressiveness	4.93 (0.44)	4.86 (0.51)	4.76 (0.66)	4.98 (0.56)	4.85 (0.49)	5.03 (0.53)	4.97 (0.61)	4.80 (0.64)	4.91 (0.56)	0.255	-

**Career motivation**											
CMQ^2 ^Intrinsic motivation	6.04 (0.51)	6.09 (0.47)	6.32 (0.42)	6.09 (0.54)	6.21 (0.40)	6.18 (0.51)	5.92 (0.59)	6.16 (0.47)	5.96 (0.61)	0.001	PSY < OS, PAED, AN < SG
CMQ^2 ^Extrinsic motivation	3.41 (0.76)	3.60 (0.76)	4.18 (0.88)	3.75 (0.81)	3.53 (0.75)	3.47 (0.72)	3.79 (0.70)	4.12 (0.87)	3.41 (0.95)	<0.001	SG, OS > rest
CMQ^2 ^Extraprofessional concerns	4.75 (0.87)	4.55 (0.94)	3.54 (1.03)	4.44 (0.95)	4.15 (1.20)	4.25 (0.83)	4.85 (1.02)	4.21 (0.89)	4.40 (1.00)	<0.001	SG < rest AN, OS, PAED < PC, PSY

**Importance of life goals (GOALS)**											
Intimacy	4.63 (0.34)	4.59 (0.43)	4.41 (0.59)	4.76 (0.31)	4.59 (0.46)	4.66 (0.47)	4.50 (0.55)	4.67 (0.37)	4.58 (0.49)	0.016	SG < GO
Affiliation	3.50 (0.75)	3.64 (0.69)	3.46 (0.92)	3.77 (0.79)	3.64 (0.68)	3.73 (0.76)	3.53 (0.73)	3.65 (0.69)	3.62 (0.73)	0.559	-
Altruism	3.73 (0.70)	3.61 (0.58)	3.73 (0.77)	3.58 (0.56)	3.71 (0.79)	3.96 (0.71)	3.41 (0.43)	3.55 (0.73)	3.66 (0.74)	0.40	-
Power	2.53 (0.76)	2.77 (0.77)	3.07 (0.86)	2.99 (0.63)	2.63 (0.74)	2.68 (0.70)	2.97 (0.72)	2.85 (0.76)	2.70 (0.81)	0.006	PC < GO, SG
Achievement	4.03 (0.56)	4.22 (0.49)	4.45 (0.46)	4.25 (0.47)	4.34 (0.48)	4.33 (0.52)	4.21 (0.46)	4.32 (0.48)	4.19 (0.56)	0.002	PC < OS, PAED, AN, SG
Variation	3.80 (0.69)	3.66 (0.70)	3.82 (0.67)	3.73 (0.75)	3.86 (0.68)	3.84 (0.66)	3.43 (0.70)	3.75 (0.75)	3.75 (0.80)	0.301	-

^1^PAQ Personal Attributes Questionnaire

^2^CMQ Career Motivation Questionnaire

PC = Primary Care, IM = Internal Medicine, SG = Surgery, GO = G&O, AN = Anaesthesiology, PAED = Paediatrics, PSY = Psychiatry, OS = Other specialities, ND = Not decided

**Table 5:** Influence of gender, personality traits, career motivation and importance of life goals on speciality choice at T_3 _(n = 522), multivariate analyses

	**Wilk's Lambda**	**F (df_effect_, df_error_)**	**p**	**Eta-quadrat**
Gender		F(8,513) = 9.14	<0.001	0.13
Personality traits	0.85	F(32,1868) = 2.65	<0.001	0.04
Career motivation	0.79	F(24,1474) = 5.34	<0.001	0.08
Life goals	0.81	F(48,2503) = 2.31	<0.001	0.04
Gender (covariates: personality traits, career motivation, life goals)		F(8,493) = 5.45	<0.001	0.08
Personality traits (covariates: career motivation, life goals)	0.92	F(32,1823) = 1.29	0.128	0.02
